# Engineering the tumor immune landscape: Translating non‐invasive physical stimulation into tumor‐associated macrophage‐targeted cancer immunotherapy

**DOI:** 10.1002/btm2.70126

**Published:** 2026-02-25

**Authors:** Tingyu Zhang, Jie Lan, Wangrui Peng, Huai Yang, Yiyang Huang, Linyuan Jin, Meng Du, Zhiyi Chen

**Affiliations:** ^1^ Department of Ultrasound, Department of Medical Imaging, The Affiliated Changsha Central Hospital, Hengyang Medical School University of South China Changsha China; ^2^ Key Laboratory of Medical Imaging Precision Theranostics and Radiation Protection, College of Hunan Province, The Affiliated Changsha Central Hospital, Hengyang Medical School University of South China Changsha China; ^3^ Institute of Medical Imaging, Hengyang Medical School University of South China Hengyang China; ^4^ Institute for Future Sciences University of South China Hengyang China; ^5^ The Seventh Affiliated Hospital, Hunan Veterans Administration Hospital, Hengyang Medical School University of South China Changsha Hunan China

**Keywords:** immunotherapy, physical therapy, polarization, tumor, tumor‐associated macrophage

## Abstract

Tumor‐associated macrophages (TAMs) shape the tumor microenvironment through plastic transitions between pro‐inflammatory M1‐like and immunosuppressive M2‐like states, yet clinical drug therapies are limited by toxicity, resistance, and delivery barriers. This review explains how non‐invasive physical stimulation (NIPS) reprograms TAMs via defined couplings between physical inputs and signaling pathways. Hypoxia‐tolerant photodynamic strategies and mild photothermal heating reset hypoxia‐ and lactate‐driven programs; cavitation‐dominant ultrasound and sonodynamic therapy trigger danger signaling and reactive oxygen species; ultrasound microbubble destruction provides endothelial repair cues; nanosecond pulsed electric fields activate cyclic GMP‐AMP synthase–stimulator of interferon genes (cGAS–STING) pathway; piezoelectric materials convert mechanical input into calcium‐dependent transcription; and appropriately dosed radiotherapy elicits immune‐active responses while avoiding hypoxia‐driven M2 recruitment. Across models, these regimens promote pro‐inflammatory reprogramming, normalize aberrant vasculature, and strengthen antitumor immunity while restraining immunosuppression. We synthesize parameter windows, delivery options, and combination strategies with checkpoint blockade and macrophage‐directed agents to guide the translation of NIPS into precise, low‐toxicity TAM‐targeted immunotherapy.

**Translational Impact Statement**

This review highlights how controllable non‐invasive physical stimulation techniques—such as ultrasound, light, electricity, and magnetism—can reprogram tumor‐associated macrophages to enhance antitumor immunity. By integrating engineering design parameters with immunological mechanisms, the work provides a bioengineering framework for developing precise, low‐toxicity immunotherapies, advancing the translation of physical stimulation–based tumor treatments toward clinical application.

## 1 INTRODUCTION

The tumor microenvironment (TME) is a dynamic and complex ecosystem composed of tumor cells, immune cells, stromal cells, and a variety of non‐cellular components.^1^ Within this network, the functional state of immune cells is considered a critical determinant of tumor fate. These immune cells may either suppress tumor progression through immune surveillance and elimination, or conversely, facilitate immune escape and disease advancement via immunosuppressive mechanisms.^2^ Among the immune cell population, tumor‐associated macrophages (TAMs) are notable for their numerical dominance and remarkable functional plasticity, playing a central role in shaping the tumor immune microenvironment (TIME) and driving tumor progression.^3^

Typically, TAMs can adopt distinct functional states in response to microenvironmental cues, with the most representative being the pro‐inflammatory M1 phenotype and the immunosuppressive M2 phenotype, both of which are critically involved in the regulation of the TME.^4^ This polarization is orchestrated by diverse regulatory signals—including cytokines, growth factors, metabolic and physicochemical cues, as well as pattern‐recognition pathways—that collectively determine the balance between pro‐inflammatory and immunosuppressive states.^5^ Although traditional methods such as chemotherapy, immune checkpoint inhibitors (ICIs) and gene therapy have shown potential in regulating TAM polarization, their clinical application is still limited by issues such as systemic toxicity, risk of drug resistance and delivery efficiency.^6^, ^7^, ^8^, ^9^, ^10^ Interestingly, non‐invasive physical stimulation (NIPS) approaches—including ultrasound, light, electricity, magnetism, and radiation—have attracted increasing attention as emerging immunomodulatory strategies. Unlike drugs or vectors that must traverse stromal barriers and maintain systemic exposure, NIPS delivers controllable energy to the tumor, offers millimeter‐level penetration, and can be repeated with adjustable parameters (intensity, frequency, duration) while preserving tissue architecture. These features directly address two bottlenecks in TAM targeting: inefficient delivery to hypoxic, acidic, and densely stromalized niches, and the need for spatially confined, reversible reprogramming.^11^, ^12^, ^13^, ^14^, ^15^ Building on these advantages, at the functional level NIPS delivers three linked outcomes within the tumor: it reprograms TAMs toward pro‐inflammatory activity, normalizes aberrant vasculature and stromal architecture to facilitate immune trafficking, and strengthens antitumor immunity while restraining local immunosuppression.^16^, ^17^, ^18^

In this review, we first outline the molecular and cellular mechanisms that govern TAM polarization and highlight the distinct contributions of M1‐ and M2‐like states to the TIME. We then summarize how these polarization programs influence tumor progression through their impact on immune regulation, stromal remodeling, vascular adaptation, and niche maintenance. Finally, we discuss recent advances in NIPS strategies for TAM reprogramming, with emphasis on their mechanistic basis, immunomodulatory potential, and translational implications for cancer immunotherapy.

## 2 MECHANISMS AND FACTORS INFLUENCING TAM POLARIZATION

**FIGURE 1:**
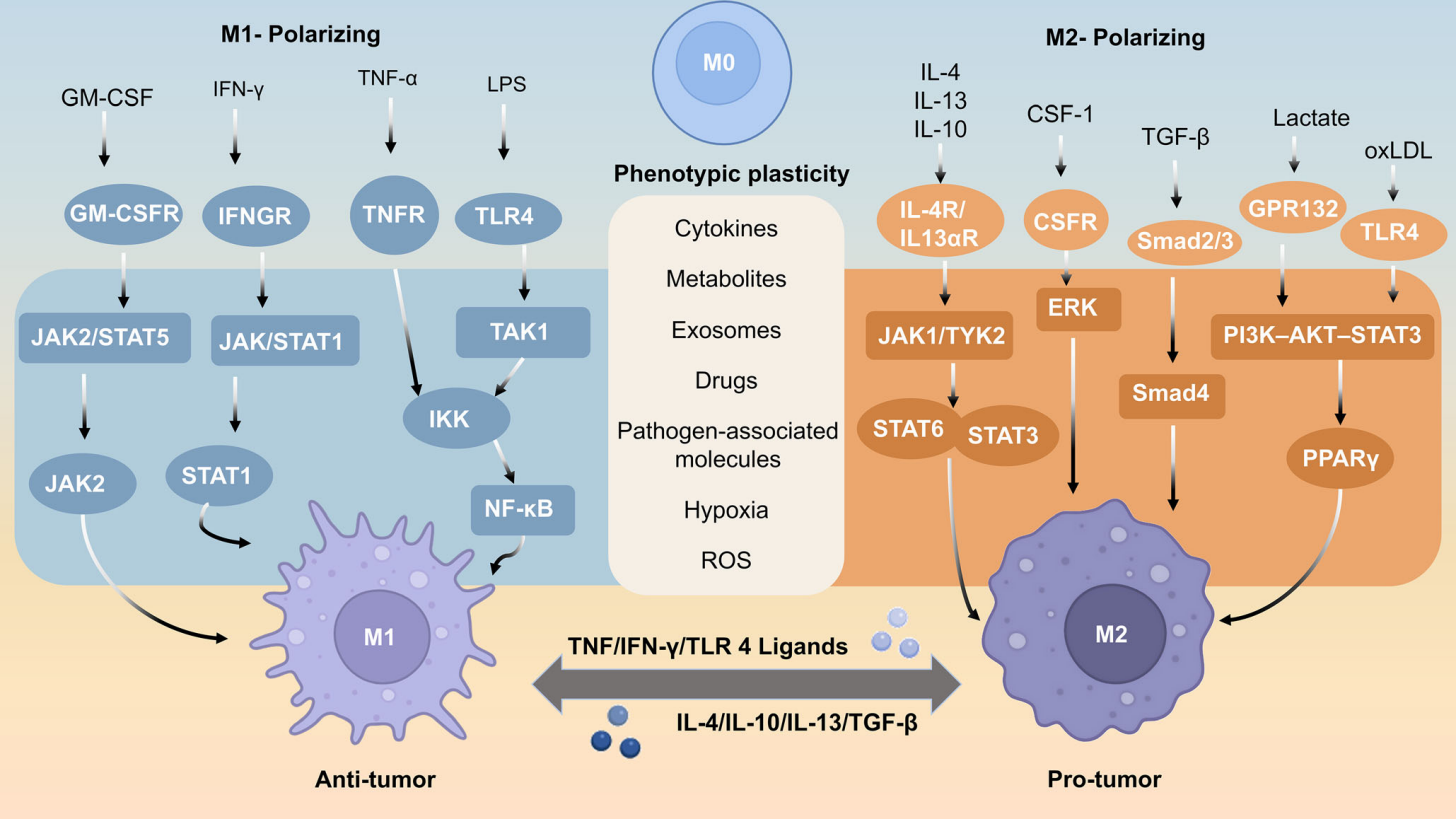
Mechanistic landscape and regulatory network governing TAM polarization in the TME. TAMs display remarkable plasticity, transitioning along a continuum from proinflammatory, M1‐like phenotypes to immunosuppressive, M2‐like phenotypes. M1 polarization is typically driven by cytokines such as IFN‐γ and TNF‐α, or microbial signals (e.g., LPS–TLR4), which activate JAK/STAT1, NF‐κB, and IRF5 pathways to enhance antigen presentation and antitumor immunity. In contrast, M2 polarization is promoted by IL‐4, IL‐10, TGF‐β, and CSF‐1 through STAT3, STAT6, PI3K/AKT/mTOR, and PPARγ signaling, fostering tissue remodeling, angiogenesis, and immune suppression. Diverse factors—including growth factors, pattern recognition receptor signals, metabolic cues, and tumor‐derived exosomes—converge on a core transcriptional network to determine TAM functional orientation within the TME. LPS, lipopolysaccharide; TAM, tumor‐associated macrophage; TLR4, Toll‐like receptor 4; TME, tumor microenvironment; TNF‐α, tumor necrosis factor‐α.

TAMs are among the most abundant innate immune cells in the TIME and exhibit remarkable plasticity, enabling dynamic transitions between a pro‐inflammatory, anti‐tumor M1‐like phenotype and an immunosuppressive, pro‐tumor M2‐like phenotype. However, this polarization is not a static binary process but rather a continuum of lineage transitions precisely regulated by multiple extracellular and intracellular signals.^19^, ^20^ Deciphering these regulatory mechanisms not only deepens our understanding of fundamental principles in tumor immunology but also identifies potential therapeutic targets for subsequent NIPS‐based targeting of TAMs (Figure 1).

### 2.1 Phenotypic functions of TAMs

M1‐like TAMs are typically induced by Type I inflammatory cues such as interferon‐γ (IFN‐γ) and tumor necrosis factor‐α (TNF‐α), as well as by Toll‐like receptor (TLR) stimulation triggered by pathogen‐associated molecular patterns (PAMPs). These exogenous signals act through the JAK/STAT1 pathway in concert with transcription factors including NF‐κB and IRF5, driving robust expression of pro‐inflammatory effectors such as iNOS, IL‐12, and TNF‐α, thereby enhancing antigen‐presenting capacity and amplifying anti‐tumor immunity.^21^

Conversely, M2‐like TAMs are typically induced by Type II immune signals such as IL‐4 and IL‐13, as well as immunosuppressive factors including IL‐10, TGF‐β, and CSF‐1. Activation of the JAK/STAT6 and STAT3 pathways, together with metabolic regulators such as PPARγ and the PI3K/AKT/mTOR axis, drives the expression of genes including Arg‐1, CD206, and Fizz1. This phenotype favors tissue repair, promotes angiogenesis, and attenuates anti‐tumor immunity.^22^, ^23^ Importantly, TAMs within tumors are rarely purely M1 or M2; rather, they exist in a dynamically balanced hybrid state continually reshaped by disease progression and microenvironmental conditions.

### 2.2 Regulators of TAM polarization

Within the TME, TAM polarization is determined by the dynamic integration of diverse regulatory signals, including cytokines secreted by immune and structural cells, PAMPs or damage‐associated molecular patterns (DAMPs), growth factors, metabolic and physicochemical cues, as well as noncanonical molecular signals. These inputs converge through distinct receptor‐mediated pathways into a limited set of transcriptional regulatory networks that shape M1‐like or M2‐like phenotypic programs (Table 1).

**TABLE 1:** Key factors and pathways regulating TAM polarization in the TME.

Category	Polarization	Stimuli/sources	Key signaling pathways	References
Cytokines and chemokines	M1	IFN‐γ, TNF‐α, GM‐CSF; CXCL9/10/11	JAK/STAT1, STAT5, NF‐κB, IRF5	24, 25, 26, 27
	M2	IL‐4, IL‐13, IL‐10, TGF‐β; CCL2/5/17/22, CXCL12	JAK1/STAT6, STAT3	
PRRs	M1	LPS (TLR4), lipopeptide (TLR2), HMGB1	MyD88/TRIF→NF‐κB/IRF	28, 29, 30, 31
	M2	Fungal β‐glucan, MMPs	CLR → STAT3/PPARγ; sustained TLR4 tolerance	
Growth factors/structural signals	M1	GM‐CSF	JAK/STAT5, NF‐κB	32, 33, 34
	M2	CSF‐1, VEGF, PDGF, EGF	CSF‐1R–PI3K/AKT/mTOR, MAPK	
Metabolic and physicochemical cues	M1	Acute hypoxia (HIF‐1α), low/moderate ROS	HIF‐1α–glycolysis, p38 MAPK	35, 36, 37, 38, 39, 40, 41, 42
	M2	Chronic hypoxia (HIF‐1/2α), lactate, acid pH (GPR65), high ROS, PGE₂, oxLDL	HIF‐2α–COX‐2/PGE₂, PPARγ, PI3K/AKT, lactate–histone lactylation	
Non‐canonical molecules and cell–cell contacts	M1	CD40L, ICAM‐1/LFA‐1, TLR agonist R848	NF‐κB, MAPK, PI3K/AKT	43, 44, 45, 46, 47
	M2	Exosomal miR‐21/222/146a, IL‐6, miR‐155‐3p, Notch‐Jagged	JAK2/STAT3, Notch intracellular domain	
Signal integration and transcriptional networks	M1	IFN‐γ, LPS, miR‐155	STAT1, NF‐κB, IRF5, p38/JNK	48, 49, 50, 51, 52, 53
	M2	IL‐4, IL‐10, TGF‐β, CSF‐1, miR‐146a	STAT3, STAT6, PPARγ, PI3K/AKT/mTOR	

Abbreviations: CLR, C‐type lectin receptor; HMGB1, high‐mobility group box 1; LPS, lipopolysaccharide; MMP, metalloproteinase; oxLDL, oxidized low‐density lipoprotein; PDGF, platelet‐derived growth factor; PRRs, pattern‐recognition receptors; TME, tumor microenvironment; TNF‐α, tumor necrosis factor‐α; VEGF, vascular endothelial growth factor.

#### 2.2.1 Cytokine and chemokine networks

TAM polarization is strongly guided by cytokines and interactions with other immune cells. Pro‐inflammatory mediators such as IFN‐γ, TNF‐α, and GM‐CSF drive M1‐like polarization through activation of the JAK/STAT1, STAT5, and NF‐κB signaling pathways, whereas IL‐4 and IL‐13 promote, and IL‐10 together with TGF‐β helps maintain, an M2‐like phenotype via STAT6 and STAT3 signaling.^24^, ^25^, ^26^ Chemokines such as CXCL9, CXCL10, and CXCL11 typically promote M1‐like polarization, whereas CCL2, CCL5, and CXCL12 facilitate the infiltration of regulatory T cells (Tregs) and myeloid‐derived suppressor cells (MDSCs), thereby indirectly reinforcing an M2‐like bias.^27^ The combined actions of these cytokines and chemokines consequently shape the abundance, spatial distribution, and polarization trajectory of TAMs.

#### 2.2.2 Pattern recognition receptors

TAMs can sense exogenous PAMPs and endogenous DAMPs through pattern‐recognition receptors (PRRs), thereby initiating corresponding immune responses. For example, lipopolysaccharide (LPS) activates NF‐κB and IRF via the TLR4–MyD88/TRIF axis, promoting the expression of pro‐inflammatory mediators characteristic of an M1‐like phenotype^28^; fungal β‐glucan activates STAT3 and PPARγ through C‐type lectin receptors (CLRs), driving the transcription of M2‐like‐associated genes.^29^ Interestingly, these signals display a double‐edged nature: acute TLR4 stimulation can promote M1‐like polarization, whereas chronic activation may induce immune tolerance. DAMPs such as high‐mobility group box 1 (HMGB1) exert pro‐inflammatory effects, while ECM degradation fragments mediated by metalloproteinases (MMPs) may instead favor a tumor‐promoting M2‐like response.^30^, ^31^

#### 2.2.3 Growth factors and structural cells

Tumor and stromal structural cells profoundly influence TAM function through the secretion of growth factors. CSF‐1 is a key driver of M2‐like polarization, while vascular endothelial growth factor (VEGF) indirectly modulates TAM phenotype by promoting angiogenesis and suppressing T‐cell activation. Growth factors such as platelet‐derived growth factor (PDGF) and epidermal growth factor (EGF), released by cancer‐associated fibroblasts (CAFs), further contribute to TAM remodeling via activation of the MAPK and PI3K pathways.^32^, ^33^, ^34^ These signals are often persistently upregulated during tumor progression, leading to the long‐term maintenance of an immunosuppressive microenvironment.

#### 2.2.4 Metabolic and physicochemical cues

The metabolic status and physicochemical microenvironment of the tumor niche profoundly influence TAM polarization. Hypoxia can upregulate key glycolytic enzymes such as HK2 and PFKFB3 via hypoxia‐inducible factor 1α (HIF‐1α) and inducible nitric oxide synthase (iNOS) to accelerate the production of nitric oxide (NO) and reactive oxygen species (ROS), thereby sustaining anti‐tumor effects. At the same time, HIF‐2α drives the expression of VEGF and other pro‐angiogenic factors associated with M2‐like polarization, promoting angiogenesis and immune suppression.^35^, ^36^ Lactate accumulation not only promotes M2‐like polarization through GPR132/GPR81 signaling but also induces histone lactylation, directly altering gene transcription.^37^, ^38^ In addition, lipid metabolic reprogramming, such as activation of the PGE₂–PPARγ pathway, has been shown to enhance the immunosuppressive function of M2‐like macrophages.^39^ Meanwhile, the acidic TME (pH ≈ 6.5) and elevated ROS create a complex bidirectional regulation: moderate ROS can act as signaling cues that support M1‐like activation, whereas sustained oxidative stress—together with tumor acidosis—engages stress‐kinase pathways (including p38 MAPK) and is linked to the induction of immunosuppressive effector modules and pro‐angiogenic cues, thereby favoring an M2‐like transition.^40^, ^41^, ^42^

#### 2.2.5 Noncanonical signaling pathways and direct intercellular communication

In recent years, beyond metabolic cues and soluble cytokines, tumor‐derived exosomes (TDEs) carrying miRNAs, proteins, and lipids have emerged as noncanonical signaling cues capable of remotely reprogramming macrophage phenotypes.^43^ For example, TDEs enriched in miR‐21 and miR‐146a can suppress M1‐like–associated pathways while upregulating M2‐like gene expression.^44^, ^45^ Likewise, Notch–Jagged contact signaling promotes M2‐like polarization, whereas CD40–CD40L interactions can restore a pro‐inflammatory M1‐like phenotype.^46^, ^47^

#### 2.2.6 Signal integration and transcriptional networks

Under the combined action of multiple exogenous stimuli, TAM polarization ultimately converges on key signaling nodes such as JAK/STAT, NF‐κB, IRFs, PPARγ, and PI3K/AKT/mTOR, with extensive crosstalk occurring among these pathways.^48^, ^49^, ^50^ For example, STAT3 and NF‐κB exhibit antagonistic activity under certain conditions, while PI3K/AKT can negatively regulate TLR‐driven pro‐inflammatory signaling.^51^ Epigenetic regulation, such as by miR‐155 and miR‐146a, can further stabilize or reverse the polarization state on this basis.^52^, ^53^ It is precisely this multilayered integration and dynamic equilibrium that endows TAMs with highly plastic functional states across different tumor types, spatial contexts, and disease stages. At the same time, it provides multiple potential targets for future repolarization strategies.

In summary, TAM polarization is a dynamic process jointly driven by cytokine networks, pattern‐recognition signaling, metabolic status, and the physicochemical microenvironment. Notably, many of these regulatory nodes function as biological interfaces that are highly sensitive to physical inputs: membrane receptors can undergo conformational changes in response to mechanical forces or electric fields; metabolic pathways respond to local physicochemical conditions such as temperature, ROS, and pH; and hypoxic or acidic niches can be modulated by external energy forms such as light, ultrasound, or magnetic fields. This implies that NIPS can act not only as an auxiliary modulator within conventional immune signaling networks but also as a precise trigger capable of reshaping TAM polarization independent of chemical mediators. Such insights provide a critical theoretical foundation for incorporating NIPS into future strategies for TAM regulation.

## 3 IMPACT OF MACROPHAGE POLARIZATION ON THE TME

**FIGURE 2:**
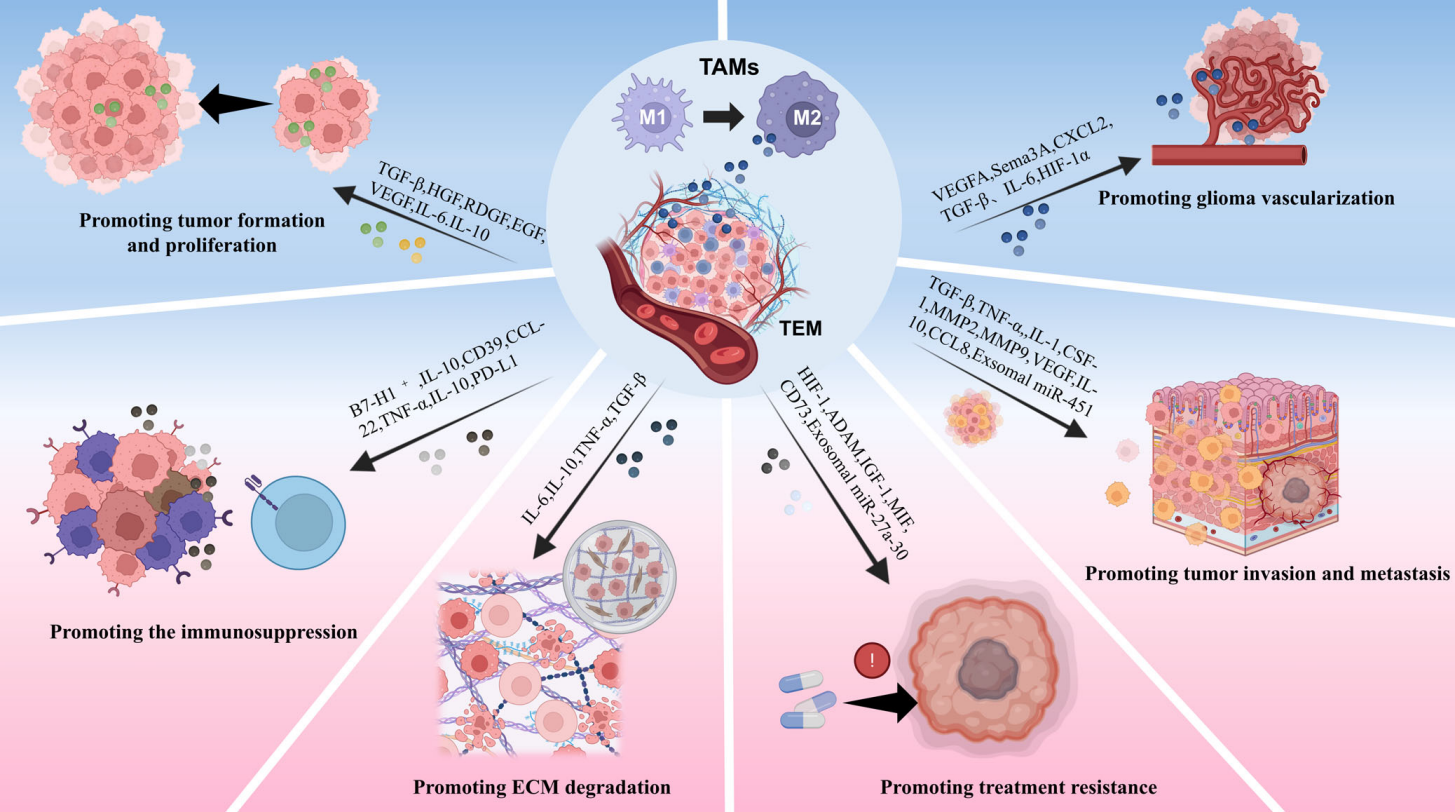
Pro‐tumoral functions of M2‐like TAMs within the TME. M2‐like TAMs orchestrate multiple tumor‐promoting processes through distinct effector molecules and signaling pathways. (1) Tumor growth is supported by secretion of EGF, transforming TGF‐β, and PDGF, which stimulate proliferation and survival of tumor cells. (2) Tumor invasion and metastasis are facilitated by MMPs and other proteolytic enzymes that degrade the extracellular matrix and enable cancer cell dissemination. (3) Tumor angiogenesis is driven by VEGF, bFGF, and IL‐8, promoting neovascularization to sustain tumor expansion. (4) Immune escape is enhanced via immunosuppressive mediators such as IL‐10 and TGF‐β, which inhibit CTLs and NK cells, and foster recruitment of Tregs and MDSCs. Collectively, these activities position M2 TAMs as central regulators of tumor progression and potential therapeutic targets for reprogramming strategies. CTLs, cytotoxic T lymphocytes; MDSCs, myeloid‐derived suppressor cells; MMPs, metalloproteinases; NK, natural killer; PDGF, platelet‐derived growth factor; TAM, tumor‐associated macrophage; TME, tumor microenvironment; VEGF, vascular endothelial growth factor.

TAMs not only participate directly in tumor immune responses as part of the innate immune system but also reshape the internal tumor ecosystem through phenotypic transitions. In early‐stage tumors, M1‐like TAMs predominate and restrict tumor expansion via pro‐inflammatory responses. As tumor progression advances, however, the M1/M2 ratio gradually declines, and immunosuppressive M2‐like TAMs become dominant, with this M2 subset further subclassified into M2a, M2b, M2c, and M2d on the basis of inducing stimuli and effector programs, thereby driving angiogenesis, stromal remodeling, metabolic adaptation, and the establishment of a pre‐metastatic niche. This dynamic phenotypic shift positions TAMs as a central hub for multilevel signal integration and microenvironmental remodeling within the TME (Figure 2).

### 3.1 Immune cell remodeling

At the immune‐cell level, TAMs in different polarization states exhibit markedly opposing functional patterns. M1‐like macrophages secrete CXCL9, CXCL10, and CXCL11 to recruit CD8^+^ cytotoxic T lymphocytes (CTLs) and natural killer (NK) cells, establishing a pro‐inflammatory amplification loop centered on IFN‐γ and TNF‐α that markedly enhances anti‐tumor immunity.^54^, ^55^ In multiple clinical cancer cohorts, the extent of M1 infiltration within tumor tissues has been positively correlated with longer overall survival.^56^, ^57^ Conversely, M2b and M2c TAMs secrete immunosuppressive factors such as IL‐10 and TGF‐β, express PD‐L1 and B7‐H4 to dampen T‐cell receptor signaling, and recruit Tregs and MDSCs to establish an immunosuppressive network.^58^, ^59^, ^60^ Such a shift often signals the onset of immune escape and is associated with poor responsiveness to both chemotherapy and ICIs.

### 3.2 Interactions with stromal cells and ECM remodeling

The interaction between TAMs and CAFs is critical for structural remodeling of the TME. M2d TAMs induce and sustain CAF activation through factors such as TGF‐β and IL‐10, while activated CAFs, in turn, secrete GM‐CSF and IL‐6 to stabilize the M2 phenotype, thereby establishing a positive feedback loop.^61^, ^62^, ^63^ The resulting sustained signaling drives TAMs to release matrix metalloproteinases (MMP2, MMP7, MMP9, and others) and cathepsins, which degrade collagen and the basement membrane and generate bioactive matrix degradation fragments that promote invasion and angiogenesis.^64^ Meanwhile, TAMs predominantly of the M2a and M2c promote Type I/III collagen deposition and matrix stiffening, and these physical changes enhance tumor cell migratory potential through YAP/TAZ mechanotransduction and integrin–FAK signaling.^21^, ^65^ By contrast, the role of M1‐like TAMs in this process is largely manifested by the inhibition of ECM degradation and CAF activation, thereby preserving tissue barrier integrity.^66^

### 3.3 Vascular and lymphatic remodeling

The vascular and lymphatic systems are critical conduits for sustaining tumor nutrient supply and facilitating metastasis, and TAMs play a central regulatory role in their remodeling.^67^ Under hypoxia, M2d TAMs stabilize HIF‐1α, upregulating VEGF and angiopoietin‐2 (ANG2) while secreting MMP9 to degrade basement membranes—facilitating endothelial cell migration and angiogenic sprouting.^68^, ^69^ This is particularly evident in Tie2‐expressing monocytes/macrophages (TEMs), which are recruited to perivascular sites by endothelial‐derived ANG2 and directly promote capillary formation.^70^ In patients with HCC, chronic lymphocytic leukemia, and ovarian cancer, TEM density correlates strongly with microvessel density.^71^, ^72^ In addition, M2d TAMs can promote lymphangiogenesis through VEGF‐C/D–VEGFR3 signaling, thereby establishing structural pathways for the pre‐metastatic niche.^73^ By contrast, M1 TAMs inhibit endothelial proliferation via CXCL9 and CXCL10 and employ ROS and NO to disrupt aberrant vasculature, thereby diminishing the tumor blood supply.^74^

### 3.4 Metabolic and physicochemical reprogramming

The physicochemical and metabolic features of the TME not only influence TAM polarization but are reciprocally reshaped by TAM activity.^39^ M2a and M2d TAMs favor lipid oxidation, tolerate high lactate levels, and thrive in an acidic microenvironment.^5^ Lactate promotes the expression of M2‐associated immunosuppressive genes through GPR132 signaling and, in cooperation with HIF‐1α, induces VEGF and Arg1 to sustain angiogenesis. Lipid metabolic products such as PGE₂ and oxidized low‐density lipoprotein (oxLDL) can likewise reinforce the M2a phenotype through the PPARγ pathway.^75^, ^76^, ^77^ M1‐like TAMs, by contrast, rely on HIF‐1α‐driven glycolysis and robust generation of ROS and NO, causing mitochondrial and redox system damage in tumor cells.^78^ This bidirectional interplay between metabolism and immunity positions TAMs as key regulatory nodes linking local metabolic homeostasis with inflammatory signaling.

### 3.5 Niche maintenance and remodeling

TAMs play a central role in maintaining and shaping the TME niche. At the niche level, they establish stable interactions with cancer stem cells (CSCs) and components of the nervous system. M2c TAMs sustain CSC stemness and migratory capacity through CCL2–AKT/β‐catenin or TGF‐β1–SMAD2/3 signaling, and in certain tumors secrete neurotrophic factors that promote nerve fiber infiltration.^79^, ^80^ Neurotransmitters released from nerve endings, in turn, enhance the immunosuppressive properties of TAMs, establishing a neuro‐immune positive feedback loop.^81^ By contrast, M1‐like TAMs tend to suppress CSC activity and disrupt the pro‐tumor influence of the neural microenvironment.^82^

Overall, TAM polarization shapes the structure and function of the TME on multiple levels by influencing immune cell activity, remodeling the extracellular matrix, regulating vascular and lymphatic networks, altering local metabolic and physicochemical conditions, and maintaining specialized niches. This broad and multifaceted regulation positions TAMs as critical nodes connecting diverse TME subsystems, implying that any intervention capable of precisely modulating their phenotype or local operating conditions could remodel the TME on multiple fronts. The following discussion will focus on how NIPS can target these key processes and offer new opportunities for TAM reprogramming.

## 4 REGULATION OF TAM POLARIZATION BY NIPS

**FIGURE 3:**
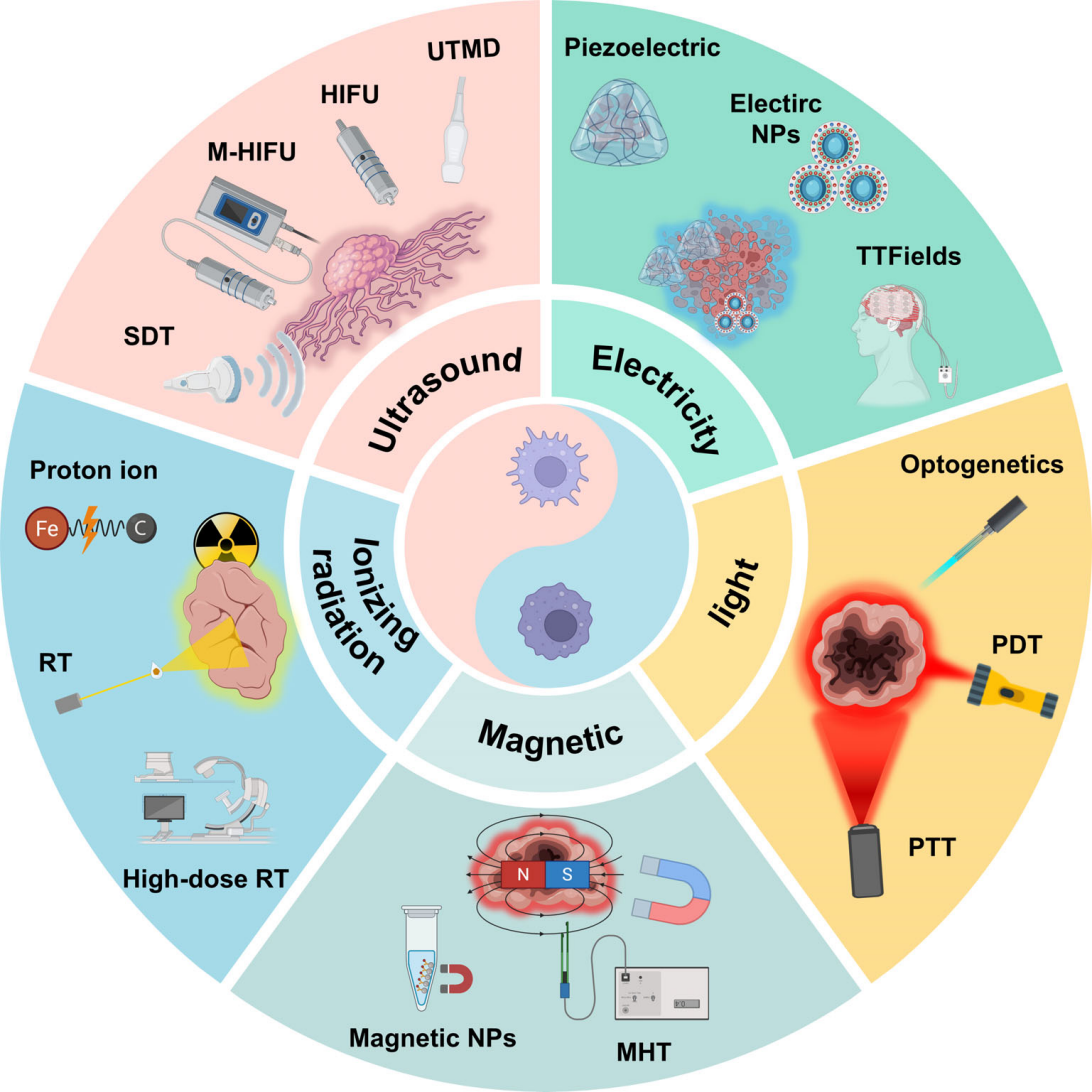
Non‐invasive physical stimulation modulates TAM polarization. Non‐invasive physical stimuli, including electrical, light, magnetic, ionizing radiation, and ultrasound stimulation, can regulate TAM polarization from a pro‐tumoral M2‐like phenotype toward an antitumoral M1‐like phenotype. These stimuli act through mechanosensitive receptors, ion channels, or metabolic reprogramming, triggering downstream signaling cascades that reshape the TME and enhance immunotherapy efficacy. TAM, tumor‐associated macrophage.

As outlined earlier, conventional strategies—such as cytokine administration and ICIs—can, to some extent, modulate TAMs activity but remain inherently constrained by systemic toxicity, off‐target effects, and limited penetration into the TME.^83^, ^84^ In contrast, NIPS has attracted growing attention in tumor immunology and bioengineering due to its unique advantages, including the absence of chemical carriers or genetic modification, tunable stimulation parameters, localized precision, and compatibility with diverse therapeutic platforms. The physical cues delivered by NIPS—such as mechanical forces, light, ultrasound, electrical and magnetic fields, or radiation—are typically detected by primary cellular sensors, including mechanosensitive structures, photosensitive receptors, and ion channels located at the plasma membrane. These cues are rapidly transduced into intracellular biochemical signals, triggering changes in ionic fluxes, alterations in second messenger dynamics, and the activation of downstream signaling cascades.^85^ Eventually, these processes converge on transcriptional reprogramming, leading to functional polarization of TAMs toward either a pro‐inflammatory (M1‐like) or immunosuppressive (M2‐like) state.^86^ It should be noted that the effects of NIPS on macrophage polarization remain difficult to define within a single mechanistic model. This is partly due to the extensive overlap and crosstalk among signaling pathways governing TAM polarization, and partly because responses are strongly dependent on stimulation parameters, such as intensity, frequency, and duration, as well as on the cellular context and physiological state of the host. As a result, identical physical cues may elicit divergent—even opposing—polarization outcomes across different experimental settings. At present, a systematic synthesis and comparative analysis of the underlying mechanisms by which NIPS regulates TAM behavior in the TME is still lacking, which to some extent limits both its predictability and translational potential (Figure 3 and Table 2).

**TABLE 2:** Summary of non‐invasive physical stimulation strategies for TAM polarization.

Stimulus type	Modality	Core mechanism	Polarization outcome	Key functional effects	References
Light	PDT—Type II (O₂‐dependent)	^1^O_2_ ROS from PS photoexcitation → ICD → DC activation; limited efficacy in hypoxia	Often maintains M2 under hypoxia	Direct tumor kill; weak immune activation under low O_2_	88
	PDT—Type I (hypoxia‐tolerant)	•O₂^−^/•OH radicals + catalytic O₂ from tumor H₂O₂ → ↓HIF‐1α → cytokine shift	M2 → M1	Relieves hypoxia; enhances PDT; increases M1 TAM infiltration	91, 92, 93
	PDT—Type III (oxygen‐independent)	Direct excitation energy transfer to biomolecules (RNA) → ICD	M2 → M1	Effective in severe hypoxia; boosts DC/T‐cell activation	94, 95
	Polarized light PBM/PDT	Alters membrane receptor conformation and ROS distribution; transcriptional modulation	In vitro: pro‐inflammatory modulation	Potential precise immune modulation without PS	96
	PTT—high temperature (>50°C)	Coagulative necrosis; HSP release	Variable (context‐dependent)	Rapid ablation; may cause collateral tissue damage	99
	PTT – mild (42–45°C)	Improved perfusion; hypoxia relief; NF‐κB/STAT1 activation; HSP70 inhibition	M2 → M1	Immune activation; synergy with ICB; reduced recurrence	100, 101, 102
	Optogenetics	Light‐controlled NF‐κB/MAPK/IRF5 activation via expressed photoreceptors	Tunable M1/M2	Programmable macrophage phenotype modulation	105, 106, 107, 108
Ultrasound	HIFU—thermal	Local hyperthermia → HSP/TAA release → DC activation	M2 → M1	Tumor ablation; systemic antitumor immunity	111, 112, 113
	M‐HIFU	Cavitation → tissue fragmentation and DAMP release (CRT, HMGB1)	M2 → M1	Immune priming; less heat damage	111, 114, 115
	UTMD	Microbubble cavitation → endothelial disruption/repair cues	M2 → M1	Increased vascular permeability; enhanced drug/ICI delivery	120, 121, 122, 123, 124, 125
	SDT	US‐triggered ROS from sonosensitizers → ICD; NF‐κB↑, cytokines↑	M2 → M1	Cytotoxicity plus durable immune reprogramming	126, 127, 128, 129, 130
Electrical	NPS	Membrane/organelle nanopores → Ca^2+^ flux, ER stress, ROS; cGAS–STING activation	M2 → M1	In situ vaccination; T/NK cell activation	138, 139, 140, 141, 142, 143, 144
	TTFields	Alternating EF‐induced mitotic disruption + ICD → NF‐κB/MAPK	Partial M2 → M1	Cytostasis; immune potentiation	148, 149, 150, 151
	Piezoelectric modulation	Mechanical → electric potential → Ca^2+^ influx; piezocatalytic ROS	M2 → M1	Local self‐powered TAM reprogramming	152, 153, 154, 155
Magnetic	MHT	Mild heat + Fe ion release → Fenton ROS	M2 → M1	Tumor cytotoxicity; immune activation	163, 164, 165, 166, 167
	Magnetic NPs (non‐thermal)	Acid‐triggered Fe^2+^ release → oxidative stress	M2 → M1	Intrinsic Fe‐mediated immune reprogramming	170, 171, 172
Ionizing radiation	RT—moderate dose (2–10 Gy)	DAMP/HMGB1, ROS, NF‐κB↑ → pro‐inflammatory cytokines↑	M2 → M1	Immunostimulation; radiosensitization	174, 175, 176
	RT—high dose (>10 Gy)	Hypoxia↑; HIF‐1α–SDF‐1/CXCR4 axis → M2 recruitment	M1 → M2	Angiogenesis; immune escape	177, 178
	FLASH‐RT	Ultra‐high dose rate; preserves normal tissue; ↑ M1/M2 ratio	M2 → M1	Potentiates CD8^+^ T‐cell synergy	186

Abbreviations: ER, endoplasmic reticulum; HIFU, high‐intensity focused ultrasound; ICI, immune checkpoint inhibitor; M‐HIFU, mechanical‐HIFU; MHT, magnetic hyperthermia; NPs, nanoparticles; NPS, nanosecond pulsed stimulation; PDT, photodynamic therapy; PTT, photothermal therapy; RT, radiotherapy; SDT, sonodynamic therapy; TTFields, tumor treating fields; UTMD, ultrasound‐targeted microbubble destruction; TAM, tumor‐associated macrophage.

### 4.1 Light

Phototherapy, encompassing photodynamic therapy (PDT) and photothermal therapy (PTT), has evolved into a non‐invasive strategy with distinct advantages, including minimal invasiveness, high spatiotemporal precision, and limited collateral damage to normal tissues. Its therapeutic effects predominantly rely on photophysical processes initiated upon excitation of photosensitizers (PSs) or photothermal agents, leading to the generation of ROS or localized hyperthermia to induce tumor cell death.^87^, ^88^, ^89^ Beyond its direct cytotoxicity, phototherapy has increasingly been recognized as an important means of TME modulation. In particular, its ability to drive TAM repolarization from a tumor‐promoting M2 phenotype toward a tumoricidal M1 phenotype highlights its unique role in bridging local physical intervention with systemic immune activation, offering a novel macrophage‐centered immunomodulatory approach.^90^

#### 4.1.1 Photodynamic therapy

PDT exerts its antitumor effects via ROS production upon photoactivation of PSs, inducing apoptosis and necrosis, and triggering immunogenic cell death (ICD) alongside TME remodeling to modulate TAM function. Conventional Type II PDT is oxygen‐dependent and thus limited under hypoxic conditions, where it may even exacerbate hypoxia and maintain the M2 phenotype.^88^ Emerging Type I PDT platforms and oxygen‐generating nanocatalysts overcome this limitation by producing highly reactive radicals (·O₂^−^, ·OH) under low‐oxygen conditions and catalyzing excess H₂O₂ to generate O₂ in situ—thereby alleviating hypoxia, suppressing HIF‐1α signaling, and favoring M2‐to‐M1 repolarization.^91^ For example, Yang et al. developed polyethylene pyrrolidone‐modified BiFeO_3_/Bi_2_WO_6_ heterojunction nanoparticles, which, under 660 nm irradiation, efficiently separated photo‐induced electron–hole pairs, enabling Type I PDT while simultaneously generating O_2_ from tumor‐associated H_2_O_2_.^92^ This dual synergy of ROS generation and oxygen supplementation enhanced tumoricidal ROS levels and created a pro‐M1 immune milieu. Similarly, in the Ce6@MnO_2_ nanoplatform, light irradiation triggers Mn^2+^‐mediated catalytic decomposition of H_2_O_2_ to generate O₂, thereby enhancing PDT efficacy and promoting the infiltration of M1‐like TAMs.^93^ In addition, oxygen‐independent Type III PDT has attracted increasing attention, as it employs PSs capable of directly transferring excitation energy to biological macromolecules such as RNA, thereby exerting cytotoxic and immunomodulatory effects even under severely hypoxic conditions.^94^ Yao et al. developed a family of RNA‐targeting PSs (NBEX, X = S, Se, Te), which achieved efficient tumor ablation and inhibition of metastasis in vivo.^95^ Among them, NBESe exhibited the most pronounced therapeutic performance, not only suppressing tumor progression but also promoting dendritic cell maturation and enhancing antitumor immune responses, highlighting the potential of Type III PDT to overcome hypoxia‐associated resistance, which may open avenues to explore TAM polarization reprogramming within the immunosuppressive TME.

Notably, beyond the typical Types I, II, and III photochemical reactions, alternative light stimulation modes can also modulate the polarization state of TAMs within the TME by affecting cell signaling pathways, metabolic status, and redox balance. In recent years, polarized light—characterized by its specific orientation of electromagnetic wave oscillation—has been considered to have potential immunomodulatory advantages in both PDT and photobiomodulation (PBM). Compared with non‐polarized light, polarized light may more effectively modulate the interaction between photon energy and cellular membranes, biomacromolecules, and mitochondrial respiratory chain components, thereby altering the spatiotemporal distribution of ROS, the conformation of membrane receptors, and downstream signaling networks, all of which are closely associated with macrophage polarization. Existing studies have demonstrated that polarized light PBM can directly alter macrophage‐related functions without the need for a PS. For instance, in the human monocytic cell line U937, exposure to polychromatic polarized light for 6 h resulted in marked modulation of receptor expression: CD14, MHC‐I, and CD11b were downregulated, whereas the costimulatory molecule CD86 was significantly upregulated. At the transcriptional level, the expression of IL1B, CCL2, NLR family pyrin domain containing 3 (NLRP3), and nucleotide‐binding oligomerization domain‐containing protein 1 (NOD1) was suppressed, while the inflammatory inhibitor NFKB inhibitor alpha (NFKBIA) and the innate immune receptor TLR9 were upregulated. NFKBIA is a key negative regulator of cytokine production, IL1B and CCL2 are important pro‐inflammatory cytokines, and NLRP3 and NOD1 are critical pattern‐recognition receptors in inflammatory signaling.^96^ These findings suggest that polychromatic polarized light may remodel macrophage phenotype by modulating the inflammatory signaling network.

However, it is worth noting that to date, there have been no direct studies applying polarized light—either in PBM or PDT settings—to regulate the polarization of TAMs within the TME. While polarized light PBM shows promise in non‐tumor macrophage models, its translation into tumor immunity remains unexplored. Polarized light PDT, in theory, could combine the photochemical advantages of conventional PDT with the directional energy delivery of polarized light to precisely reshape the immune landscape within the TME. Significant gaps remain in this field with respect to TAM modulation: (i) insufficient clinically relevant evidence; (ii) lack of optimized combinations of wavelength, polarization degree, illumination dose, and PSs; and (iii) absence of systematic mechanistic studies. Future research should therefore focus on standardizing irradiation parameters, elucidating underlying mechanisms, and performing rigorous preclinical validation to advance the translational application of polarized light PDT in cancer immunotherapy.

#### 4.1.2 Photothermal therapy

TAM polarization is also sensitive to variations in temperature. In inflammatory contexts, such as fever, temperature elevation enhances signaling via phosphorylation of the inhibitor of the NF‐κB kinase complex, leading to NF‐κB nuclear translocation and activation of pro‐inflammatory genes such as TNF‐α. This TNF‐α expression is tightly linked to heat shock protein 70 (HSP70), whose inhibition results in reduced TNF‐α levels.^97^ The effect of hyperthermia on macrophages can be indirect as well. For example, in triple‐negative breast cancer (TNBC) models, hyperthermia‐treated MDA‐MB‐231 cells release HSPB8‐containing exosomes that promote M1 polarization in TAMs—upregulating IL‐12 and iNOS while downregulating CD206 and Arg‐1—via activation of MAPK, TNF, and IL‐17 signaling pathways.^98^

In PTT, light‐absorbing agents are used to convert near‐infrared (NIR) light into heat. High‐temperature PTT (>50–55°C) rapidly ablates tumors via coagulative necrosis but can cause nonspecific tissue injury and disproportionate inflammatory responses.^99^ By contrast, mild PTT (≈42–45°C) avoids extensive tissue destruction while improving perfusion, relieving hypoxia, and mitigating acidosis in the TME—conditions conducive to immune activation and TAM reprogramming.^100^ For example, Deng et al. showed that a graphene oxide–PEG (GO‐PEG) nanoplatform under 808 nm irradiation significantly downregulated M2 markers (CD206, Arg‐1) and upregulated M1 markers (iNOS, CD86), even under mild hyperthermia conditions.^101^ Nevertheless, mild PTT can upregulate protective HSPs, which help tumor cells withstand thermal stress.^102^ To overcome this limitation, Zhong et al. engineered pH‐responsive Ag₂S nanodots loaded with the HSP70 inhibitor quercetin, which achieved lysosome‐triggered drug release, effectively suppressed HSP70‐mediated thermotolerance, and enabled complete tumor ablation without recurrence under NIR irradiation.^103^ More recently, Ding et al. developed a polydopamine‐coated nucleic acid nanogel for siRNA‐mediated Hsp70 silencing, which robustly downregulated HSP70 expression and thereby allowed efficient low‐temperature PTT (~42–45°C) with minimized collateral damage in vivo.^104^ However, the limited light penetration depth of PTT remains a major limitation, thereby motivating its integration with immune checkpoint blockade or other therapeutic modalities to achieve deeper tissue reach and more sustained immunomodulatory effects, which is likely to represent a future trend in this field.

#### 4.1.3 Optogenetics

Optogenetics, integrating optical and genetic methodologies, offers precise spatiotemporal control of cellular signaling with reversible kinetics through the expression of genetically encoded light‐responsive proteins, including channelrhodopsins, halorhodopsins, and photoreceptor‐based signaling actuators.^105^ Initially established as a transformative approach in neuroscience, optogenetics has recently expanded into immunological settings, enabling the programmable manipulation of signaling cascades and functional states in diverse immune cell types, including macrophages. A representative example is the optogenetic assembly of IKKα and IKKβ, which triggers robust NF‐κB pathway activation in mammalian cells upon blue‐light illumination, allowing rapid induction of pro‐inflammatory transcriptional programs.^106^ NF‐κB signaling is a pivotal determinant of macrophage polarization, implicating such platforms as potential tools for TAM reprogramming in the TME.

Other signal transduction pathways relevant to macrophage polarization have also been engineered for optical control. Bugaj et al. demonstrated phytochrome‐based modulation of the Ras/Erk pathway, establishing a versatile system for dissecting MAPK signaling dynamics in mammalian cells.^107^ More directly, Pan et al. developed an optogenetic circuit targeting IRF5, a transcription factor critical for M1‐like polarization. Their system achieved light‐inducible activation of IRF5 in bone marrow‐derived macrophages, resulting in upregulated expression of M1 markers such as iNOS and TNF‐α, alongside suppression of M2‐associated genes.^108^

Given the inherent plasticity of TAMs, optogenetics presents a compelling modality to dynamically tailor macrophage phenotypes within solid tumors. Potential strategies include optical regulation of cytokine networks (e.g., TNF‐α, IL‐12, IL‐10), modulation of metabolic programs (e.g., glycolysis versus oxidative phosphorylation bias), and precise tuning of transcriptional regulators (e.g., STAT1, STAT6, IRF5) in situ. Nevertheless, translational application is currently restricted by limitations in gene delivery efficiency, tissue light penetration, and the potential immunogenicity of microbial or plant‐derived photoreceptors. Future work should focus on integrating optogenetic control elements with advanced delivery platforms—such as macrophage‐targeted nanoparticles or implantable micro‐LED arrays—and combining them with immunomodulatory therapies, including immune checkpoint blockade or photodynamic therapy. Such integration could enable spatiotemporally resolved and phenotype‐specific TAM modulation, offering a versatile avenue for next‐generation light‐based cancer immunotherapy.

Overall, light‐based stimulation offers high spatiotemporal controllability and a direct means to couple local tumor injury with immune activation, making it well suited for macrophage‐centered remodeling of the TIME. PDT can trigger ICD and danger signaling that favors pro‐inflammatory macrophage programs, but its efficacy is highly dependent on oxygen availability and light penetration, particularly in hypoxic or deeply seated lesions; hypoxia‐tolerant designs and oxygen‐supplementing strategies improve robustness while increasing platform complexity and translational burden. PTT provides temperature‐tunable intervention, with mild heating more compatible with immune reprogramming through improved perfusion and mitigation of hypoxia‐driven suppression, whereas excessive heating may induce collateral injury and stress responses that compromise durability. Optogenetics enables exceptional programmability for interrogating and controlling polarization‐relevant pathways, yet translation remains constrained by gene delivery, tissue light access, and potential immunogenicity. Collectively, clinically feasible light delivery, real‐time dosimetry of irradiation and temperature, and rational combinations with immune checkpoint blockade or macrophage‐directed agents will be essential for reproducible clinical implementation.

### 4.2 Ultrasound

Ultrasound, a non‐invasive modality with high spatiotemporal resolution, has emerged as a promising tool for modulating TAM polarization. By precisely adjusting parameters such as frequency, intensity, and exposure duration, ultrasound can selectively reprogram TAMs from the tumor‐promoting M2 phenotype toward the tumoricidal M1 phenotype.^109^ This phenotypic switch facilitates the remodeling of an immunosuppressive TME and hinders malignant progression.^110^ Notably, studies have reported that even ultrasound irradiation alone can induce M1‐like polarization of TAMs, underscoring its potential as a novel immunoregulatory intervention.^109^ Below, we summarize the effects of different ultrasound modalities on TAM regulation and discuss their respective advantages and limitations.

#### 4.2.1 High‐intensity focused ultrasound

High‐intensity focused ultrasound (HIFU) is a non‐invasive method of physical stimulation that focuses high‐energy ultrasound beams precisely onto target tissues within the body. The energy is concentrated at the focal point, generating high temperatures or mechanical effects that can destroy pathological tissues or modulate cellular physiological functions, with minimal impact on the surrounding tissue.^111^ In general, HIFU stimulation primarily achieves its effects through two mechanisms: thermal and mechanical. Thermal HIFU ablation eradicates tumors by rapidly heating targeted tissues above the necrotic threshold, resulting in protein denaturation and coagulative necrosis. During this process, the release of HSPs and tumor‐associated antigens promotes dendritic cell (DC) activation and antigen presentation, thereby amplifying tumor‐specific T‐cell responses.^112^ Existing studies have shown that HIFU significantly reduced the serum levels of immunosuppressive cytokines such as TGF‐β and VEGF, thereby partially alleviating the immunosuppression in the TME and promoting the repolarization of M2 TAMs toward the M1 phenotype.^113^ This process enhances the immune system's recognition and attack on tumor cells, thereby strengthening the inhibition of tumor immune evasion. However, thermal HIFU may trigger excessive inflammatory responses or spatially heterogeneous tissue damage. The accumulation of thermal effects in localized tissues may lead to uneven immune responses, and the high temperatures could impair the function of normal tissues, affecting the overall therapeutic outcome.

In contrast to thermal HIFU, mechanical HIFU (M‐HIFU) disrupts tumor tissues by generating high‐pressure acoustic pulses and cavitation effects, avoiding significant thermal accumulation and thereby reducing damage to surrounding healthy tissues. M‐HIFU induces tissue destruction through the rapid formation and collapse of bubbles, generating mechanical forces that cause local cellular fragmentation and release tumor antigens and DAMPs, which subsequently activate the innate immune system, trigger pro‐inflammatory cascades, and further stimulate adaptive immune responses.^111^ Recent studies have underscored the potential of M‐HIFU in inducing TAM polarization. For instance, Pahk et al. demonstrated that M‐HIFU promotes the M1 polarization of macrophages and reprograms M2 macrophages toward an M1 phenotype in human breast cancer cells. This process is mediated by the release of key DAMPs, such as calreticulin (CRT) and HMGB1, which activate immune responses and recruit immune cells to the TME.^114^ Ruger et al. further supported this by showing in vivo that M‐HIFU not only ablates tumor tissue but also induces significant immunomodulatory effects, including increased production of pro‐inflammatory cytokines and chemokines, thereby reprogramming TAMs from a pro‐tumoral M2 phenotype to an anti‐tumoral M1 phenotype and enhancing the overall anti‐tumor immune response.^115^

However, single M‐HIFU treatment is often limited by the small tumor ablation volume, and its efficacy in activating the immune system largely depends on the release of tumor antigens and immune cell infiltration, including the reprogramming of TAMs. Therefore, combining M‐HIFU with immune enhancers or ICIs can significantly enhance its therapeutic potential by modulating the TME. Eranki et al. found that when the ablation volume is less than 2%, M‐HIFU alone is insufficient to trigger an effective immune response. In such cases, ICIs are required to overcome immune suppression and enhance the anti‐tumor immune response.^116^ Similarly, Singh et al. showed in a murine melanoma model that M‐HIFU combined with immune stimulation markedly increased M1 TAM proportions and reinforced T‐cell‐mediated antitumor responses.^117^ Regarding treatment sequencing, the teams of Abe et al. and Pepple et al. have proposed more effective approaches, namely “ICI initiation after M‐HIFU” or “simultaneous initiation of M‐HIFU and ICI.^118^, ^119^” These strategies ensure that antigen release induced by M‐HIFU occurs in parallel with ICI action, promoting the polarization of TAMs and enhancing the immune system's ability to recognize and attack tumor cells, thereby improving therapeutic outcomes. Future optimization of HIFU‐specific parameters and its combination with immunotherapies holds the potential to significantly enhance the precision and long‐term effectiveness of macrophage reprogramming, paving the way for more effective cancer therapies.

#### 4.2.2 Ultrasound‐targeted microbubble destruction

Ultrasound‐targeted microbubble destruction (UTMD) employs low‐frequency ultrasound to induce cavitation of exogenous microbubbles, generating shear forces, shock waves, and microjets that transiently disrupt cell membranes and vascular endothelium, significantly improving local permeability across biological barriers. In an orthotopic pancreatic cancer model.^120^ Lin et al. demonstrated that UTMD promoted TAM phenotype switching, as evidenced by upregulation of M1 markers (CD86, iNOS, IL‐6, TNF‐α) and downregulation of M2 markers (IL‐10, Arg1).^121^ From a mechanistic perspective, endothelial perturbation induced by microbubble cavitation may influence TAM recruitment and polarization.^122^ Furthermore, the endothelial repair process could offer critical microenvironmental cues for M1‐like reprogramming.^123^ Importantly, most existing studies on UTMD‐mediated TAM regulation have been conducted in combination with immunotherapies or drug delivery systems. In such contexts, UTMD not only directly facilitates TAM repolarization but also enhances intratumoral uptake and efficacy of co‐administered agents, yielding synergistic antitumor effects superior to monotherapy. For instance, Wang et al. demonstrated that UTMD‐assisted delivery of a PD‐L1–targeted IL‐15 mRNA nanoplatform synergistically enhanced antitumor immunity, providing a strategy to strengthen TAM repolarization in combination settings.^124^ Similarly, Arrieta et al. showed that UTMD‐mediated co‐delivery of doxorubicin and anti–PD‐1 antibodies across the blood–brain barrier improved immune activation in glioma, further underscoring its potential to augment TAM repolarization when integrated with immunotherapy.^125^

#### 4.2.3 Sonodynamic therapy

Sonodynamic therapy (SDT) employs low‐intensity ultrasound to activate sonosensitizers, generating ROS and other oxidative radicals. This process not only induces direct cytotoxicity against tumor cells but also contributes to the immunological reprogramming of TAMs.^126^, ^127^ For example, chlorin e6‐mediated SDT induces ICD and releases immunostimulatory signals that promote pro‐inflammatory TAM polarization.^128^ Mechanistically, ROS activate NF‐κB signaling, upregulate proinflammatory cytokines, induce mitochondrial injury, and trigger DAMP release, collectively sustaining an M1‐favoring immune niche.^129^, ^130^

Recent synergistic strategies have integrated SDT with nanotechnology, enabling co‐delivery of sonosensitizers with targeting ligands, immunoadjuvants, or oxygen carriers to enhance local accumulation and ROS generation. For example, Gong et al. reported that hybrid cell membrane‐coated nanoparticles carrying both a sonosensitizer and oxygen donor mitigated TME hypoxia, amplified SDT‐induced ICD, and promoted durable M1 polarization in TNBC models, leading to potent antitumor and antimetastatic responses.^131^ In terms of targeting‐ligand strategies, Feng et al. developed a pH/US dual‐responsive HMME/MCC nanosystem functionalized with hyaluronic acid (HA) for CD44 targeting, which markedly improved intratumoral deposition and antitumor efficacy, thereby reinforcing SDT‐driven inflammatory reprogramming.^132^ Moreover, Zhang et al. designed a Ce6–polyphenol/Fe(II) metal–phenolic nanocomplex co‐encapsulating lactate oxidase and atovaquone to reduce lactate and oxygen consumption, amplify SDT efficacy, and mitigate metabolic cues associated with M2 polarization.^133^ Persistent hypoxia in the TME, however, remains a fundamental limitation for ROS‐dependent SDT efficacy. Future solutions may involve self‐oxygenating or oxygen‐generating nanoplatforms, catalytic oxygenation systems, and combination with PD‐1/PD‐L1 blockade or PDT, thereby overcoming hypoxia and enhancing immune modulation.

Taken together, ultrasound‐based strategies offer deep penetration and flexible parameter tuning, making them attractive for modulating TAMs in anatomically diverse tumors. Thermal HIFU provides robust local cytoreduction and antigen release but requires careful thermal control to avoid off‐target injury, whereas mechanical HIFU reduces heat accumulation yet may be limited by ablation volume and the consistency of immune priming. UTMD is particularly advantageous for enhancing intratumoral delivery and vascular modulation, but its effects depend on tumor perfusion and cavitation control, with potential risks of microvascular damage under excessive parameters. SDT can couple ROS‐driven ICD with immune remodeling in deep tissues, although hypoxia and sensitizer delivery remain major practical constraints. Collectively, standardizing acoustic parameter windows and integrating imaging‐based monitoring will be critical for reproducible translation and for rational combination with checkpoint blockade.

### 4.3 Electrical

Electrical stimulation involves the application of controlled electric fields or signals to modulate cell function by altering membrane potential and downstream signaling.^134^ While widely used in wound healing, neural/muscular regeneration, and bone repair, immune cells—including macrophages—also exhibit inherent electrophysiological properties, rendering electrical stimulation a candidate approach for regulating TAM polarization.^135^, ^136^, ^137^

#### 4.3.1 Nanosecond pulse stimulation

Nanosecond pulse stimulation (NPS), also referred to as nanosecond pulsed field ablation (nsPFA™), delivers ultra‐short, high‐intensity electrical pulses that transiently permeabilize both plasma and organelle membranes, generating nanopores that permit rapid ion flux, perturb ionic homeostasis, and initiate endoplasmic reticulum (ER) stress together with ROS production.^138^, ^139^ These biophysical events induce the release of DAMPs and trigger regulated cell death, a defining hallmark of NPS therapy.^140^, ^141^, ^142^ By enabling gradual clearance of dying cells over several days, NPS preserves the extracellular matrix and vascular networks, simultaneously providing a temporal window for the recruitment and activation of immune cells. The resulting ICD is capable of reshaping the TME into a more immunostimulatory state. Preclinical studies have consistently demonstrated the capacity of NPS to modulate the myeloid compartment within tumors. In murine models, Nanajian et al. reported a depletion of pro‐tumorigenic M2‐like TAMs, accompanied by a shift of residual macrophages toward an M1 phenotype, together with reductions in Tregs and MDSCs, thereby reinforcing a pro‐inflammatory milieu.^143^ In hepatocellular carcinoma, NPS was shown by Qian et al. to activate the cGAS–STING pathway in intratumoral CD103^+^ dendritic cells (DCs), initiating an IL‐12–NK–CCL5 feedback circuit that sustained DC recruitment and facilitated M1‐oriented TAM polarization.^144^ In pancreatic ductal adenocarcinoma, He et al. demonstrated that NPS provoked HMGB1 release, which engaged the receptor for advanced glycation end‐products–MAPK–p38 axis, driving inflammatory M1 differentiation and enhancing antigen presentation capacity.^145^ These immunomodulatory effects are intrinsically linked to the spatiotemporal precision of NPS, which confines its bioelectrical impact to the target region between bipolar electrodes, resulting in minimal off‐target injury and sparing of surrounding healthy tissues. Notably, over 5000 dermatological NPS procedures have been completed with only transient hyperpigmentation reported as a side effect, highlighting the safety profile of clinical energy delivery.^146^, ^147^ Nevertheless, translation to deep‐seated tumor indications remains technically challenging due to limitations in electrode access and uniform field coverage. Furthermore, optimal pulse parameters to maximize TAM reprogramming without collateral damage have yet to be systematically defined.

Given its ability to induce ICD while simultaneously remodeling the myeloid landscape, NPS is well‐positioned for combination with immune checkpoint blockade, metabolic reprogramming agents, or cytokine therapies. Integration with high‐dimensional immune profiling—such as single‐cell RNA sequencing and spatial transcriptomics—will be instrumental in delineating TAM subset dynamics and identifying synergistic immune circuits. Such mechanistically guided approaches may enable the rational deployment of NPS as a spatiotemporally precise modality for functional TAM reprogramming in solid tumors.

#### 4.3.2 Tumor treating fields

Treating Fields (TTFields) are low‐intensity (1–3 V/cm), intermediate‐frequency (100–300 kHz) alternating electric fields delivered non‐invasively via transducer arrays. While originally designed to inhibit tumor growth through disruption of mitotic spindle assembly and induction of mitotic catastrophe,^148^, ^149^ increasing evidence indicates that TTFields can also remodel the TIME. In the myeloid compartment, TTFields have been linked to indirect TAM reprogramming through the induction of ICD in tumor cells, characterized by CRT exposure, ATP secretion, and HMGB1 release. These DAMPs can activate pattern recognition receptors on macrophages, thereby modulating their polarization state.^150^ In line with this, Park et al. further demonstrated that TTFields augment macrophage pro‐inflammatory cytokine secretion and activate NF‐κB/MAPK pathways, thereby promoting M1‐like polarization or partially reprogramming M2 TAMs.^151^ However, direct in vivo evidence remains scarce, and further mechanistic dissection and optimization are warranted for combinatorial regimens with ICIs. Although in vitro and ex vivo studies support a role for TTFields in promoting pro‐inflammatory macrophage functions, direct in vivo evidence quantifying TAM subset redistribution and activity remains limited. Given the sensitivity of TTFields' biological effects to parameters such as frequency and intensity, optimizing these settings for immune modulation could potentiate TAM repolarization. Rational combination strategies—particularly with ICIs or modulators of macrophage metabolism—warrant systematic investigation to exploit TTFields as a non‐invasive adjunct for myeloid reprogramming in solid tumors.

#### 4.3.3 Piezoelectric modulation

The piezoelectric effect describes the bidirectional energy conversion between mechanical deformation and electric charge generation in non‐centrosymmetric materials.^152^ Piezoelectric biomaterials—including ceramics, polymers, and polymer–ceramic composites—can convert external mechanical stimuli into localized electric potentials, enabling wireless, site‐specific bioelectrical stimulation.^153^ Among these, polyvinylidene fluoride (PVDF) with a β‐phase crystalline structure exhibits strong piezoelectric coefficients, chemical stability, and favorable biocompatibility.^154^ In the context of tumor immunomodulation, Kong et al. demonstrated that ultrasonic activation of β‐PVDF films generated localized alternating potentials that directly modulated macrophage phenotype.^155^ Mechanistically, the electrical stimulation opened voltage‐gated Ca^2+^ channels, triggering Ca^2+^ influx and activating the Ca^2+^–CAMK2A–NF‐κB signaling axis, which in turn upregulated ErbB, mTOR, and HIF‐1 pathways associated with pro‐inflammatory responses. This bioelectrical cue promoted macrophage polarization toward an M1‐like phenotype, characterized by increased TNF‐α, IL‐6, and iNOS expression, while suppressing M2‐associated Arg‐1. Functionally, macrophages conditioned by β‐PVDF plus ultrasound exhibited enhanced tumoricidal activity in co‐culture assays with cancer cells.

Beyond direct bioelectric stimulation, piezoelectric materials activated by ultrasound can catalyze ROS generation through piezocatalytic band bending, accelerating O₂ to •O₂^−^ and H₂O to •OH conversion.^156^, ^157^, ^158^ In the hypoxic and acidic TME, these ROS not only induce tumor cell apoptosis but can also modulate TAM polarization, as elevated oxidative stress has been shown to promote M1 phenotypes and inhibit M2‐associated immunosuppressive programs. For example, oxygen‐vacancy‐rich BiO_2−*x*_ nanosheets integrated piezocatalysis with enzyme‐like activity under ultrasound, producing abundant ROS and displaying broad immunostimulatory potential in murine tumor models.^159^ Similar approaches using tetragonal BaTiO_3_ or ultrathin NiFe‐LDH nanosheets have demonstrated efficient ultrasound‐triggered ROS generation with favorable biocompatibility, suggesting cross‐platform applicability for TAM repolarization via oxidative and electrical cues.^160^

Overall, piezoelectric materials combined with non‐invasive activators such as focused ultrasound offer a self‐powered strategy for localized TAM reprogramming. Advantages include the ability to couple bioelectric signaling and redox modulation within the TME without surgical implantation of electrodes. While preclinical evidence—particularly direct in vivo validation of TAM subset shifts—remains limited, the integration of piezoelectric platforms with high‐resolution immune profiling could accelerate translational development toward minimally invasive, immunotherapy‐adjuvant cancer treatments.

Taken together, electrical stimulation links biophysical inputs to innate immune activation and offers multiple practical routes for macrophage reprogramming. NPS can induce immunogenic tumor cell death and engage nucleic acid–sensing inflammatory circuits to remodel the myeloid compartment, but its use in deep tumors is constrained by electrode access and the challenge of achieving uniform field coverage while sparing surrounding tissues. TTFields provide a fully non‐invasive and repeatable modality with established clinical deployment for tumor control and may elicit immunogenic stress that indirectly favors macrophage activation; however, macrophage‐centered in vivo evidence under clinically relevant conditions remains limited, and skin tolerability can restrict long‐duration treatment. Piezoelectric platforms enable wireless, localized electrical cue generation via mechanical activation, supporting calcium‐dependent transcriptional changes and redox modulation, yet translation depends on material biocompatibility, efficient tumor delivery and retention, and reproducible in vivo electrical output. Collectively, defining parameter windows that balance efficacy and tolerability and integrating rational combinations with checkpoint blockade or macrophage‐targeted agents will be important for consistent clinical scaling.

### 4.4 Magnetic

Magnetic fields possess excellent tissue penetration and controllability, making them attractive non‐invasive stimuli for tumor therapy and immunomodulation. At the cellular level, magnetic fields can modulate ion channels, membrane potential, and iron metabolism, thereby altering macrophage function.^161^ Different field parameters produce divergent outcomes: low‐frequency alternating fields show limited TAM effects, whereas moderate static or high‐gradient fields tend to promote M2 polarization and tissue repair.^162^ In tumors, combining magnetic stimulation with magnetic nanoparticles (MNPs)—capable of generating localized heat or chemical effects—offers novel avenues for TAM reprogramming.

#### 4.4.1 Magnetic hyperthermia

Magnetic hyperthermia (MHT) employs MNPs to transform alternating magnetic field (AMF) energy into localized heat within tumor tissues.^163^ While high‐temperature MHT (>50°C) achieves rapid tumor ablation, mild MHT (39–45°C) has attracted increasing interest for its ability to not only impair tumor cell viability but also remodel the TIME, particularly by reprogramming TAMs.^164^, ^165^ In this context, sublethal thermal stress can trigger the release of DAMPs, such as HMGB1, ATP, and CRT, from tumor cells, which subsequently engage macrophage pattern‐recognition receptors to promote M1‐like activation.^166^ Simultaneously, Fe‐containing MNPs can undergo partial dissolution in acidic TMEs or within lysosomes, liberating Fe^2+^/Fe^3+^ ions that catalyze Fenton or Fenton‐like reactions to produce highly reactive •OH radicals. These radicals act both as potent cytotoxic agents and as secondary messengers that promote TAM repolarization, while also contributing to ferroptosis in tumor cells. Furthermore, mild MHT amplifies oxidative stress by accelerating Fenton chemistry and inducing mitochondrial dysfunction in tumor cells, thereby elevating ROS levels and skewing the local cytokine milieu toward pro‐inflammatory phenotypes that favor M1 polarization while suppressing M2 programs.^167^ Building on these mechanisms, Hu et al. developed pH‐responsive Fe_3_O_4_ nanoparticles that preferentially aggregated in acidic regions of the TME, thereby enhancing thermal conversion efficiency and promoting TAM reprogramming via the combined effects of hyperthermic stress and Fe‐ion release.^168^ This dual‐modality intervention markedly shifted macrophage populations toward an M1 phenotype both in vitro and in vivo. Similarly, Liu et al. engineered graphene oxide‐coated magnetic nanorings that, when activated by mild MHT, catalyzed sustained ROS generation through Fenton reactions, leading to significant upregulation of M1 markers iNOS, CD86 and concomitant suppression of the M2 marker CD206.^169^ Collectively, these studies highlight a paradigm shift in MHT from a purely physical tumor‐ablative modality to a multifunctional immunomodulatory platform in which thermal cues are integrated with redox modulation and metal‐ion dynamics to influence macrophage biology. While such effects are maximized in the presence of localized hyperthermia, emerging evidence indicates that certain magnetic nanoparticles can directly modulate TAM phenotypes through their intrinsic physicochemical properties even in the absence of appreciable temperature elevation—an aspect discussed in the following section.

#### 4.4.2 Magnetic nanoparticles

Beyond their function as heat mediators in MHT, certain MNPs possess intrinsic immunomodulatory activity that operates independently of significant bulk temperature rise. In contrast to the hyperthermia‐dependent pathways described above, these effects arise primarily from the material's inherent capacity to release Fe^2+^/Fe^3+^ under acidic TME conditions, thereby perturbing macrophage iron homeostasis, inducing oxidative stress, and promoting pro‐inflammatory cytokine secretion that drives the conversion of TAMs from an immunosuppressive M2 state to a pro‐inflammatory M1 phenotype.^170^ Material parameters—including particle size, morphology, and surface chemistry—strongly influence these non‐thermal effects; for instance, particles exceeding 100 nm display enhanced propensity for TAM uptake in vivo,^171^ while anisotropic architectures with higher magnetic anisotropy can alter aggregation behavior and intracellular dissolution kinetics, modulating Fe‐ion release and redox signaling within macrophages. Surface engineering with polymers or inorganic shells provides an additional tuning lever, allowing particles to be optimized either for immune evasion and prolonged circulation or, conversely, for selective capture by macrophages as delivery vectors.^172^ Upon intratumoral accumulation, localized nanoparticle clustering amplified magnetothermal conversion under AMF exposure and simultaneously reinforced pro‐inflammatory signaling within both the carrier macrophages and neighboring TAM populations. Such cell‐based delivery strategies not only improve MNP enrichment in tumors but also potentiate immune reprogramming effects, offering a non‐invasive and thermally synergistic avenue for targeted TAM modulation within complex TMEs.

Overall, magnetic–based strategies offer deep tissue penetration and external controllability, while MNPs enable magnetic inputs to be converted into local thermal and redox cues for TAM modulation. MHT can couple mild hyperthermia with DAMP release and Fe‐related ROS amplification to support pro‐inflammatory reprogramming, but its performance depends on sufficient intratumoral MNP deposition and controllable heating under AMF, with thermal heterogeneity and off‐target accumulation as key constraints. Beyond heating, MNPs may also drive non‐thermal TAM reprogramming via Fe release and redox/iron‐homeostasis perturbation, yet these effects are highly material‐ and context‐dependent and raise concerns regarding biodistribution and clearance. Collectively, improving delivery control and standardizing particle characterization and safety evaluation will be important for reproducible translation.

### 4.5 Ionizing radiation

Ionizing radiation (IR) underpins radiotherapy (RT), a cornerstone of cancer management. Beyond direct cytotoxicity via DNA damage, RT shapes the TIME in a dose‐dependent manner, with profound effects on TAMs. Low‐dose RT (often ≤2 Gy per fraction) has been reported to elicit anti‐inflammatory macrophage programs in certain settings, although TAM responses in the TIME are highly context‐ and regimen‐dependent.^173^ Moderate doses (2–10 Gy) generally promote M1‐like activation, as shown by Stary et al. in rectal cancer biopsies where 2 Gy reduced CD163 while increasing TNF‐α^+^/iNOS^+^ TAMs.^174^ Such responses involve DAMP/HMGB1 signaling, NOX2‐ROS‐ATM activation, and NF‐κB p65 phosphorylation, leading to elevated TNF‐α, IL‐1β, IL‐6, and IL‐12 expression.^175^, ^176^ High doses (>10 Gy), especially hypofractionated regimens, often exacerbate hypoxia, activating the HIF‐1α–SDF‐1/CXCR4 axis and recruiting radioresistant M2‐like TAMs, which promote angiogenesis via VEGF and mediate immune escape through upregulation of IL‐10, Arg1, and PD‐L1.^177^, ^178^ CAF responses and radiation‐induced extracellular vesicles can further drive M2 polarization via CCL2 or glycolytic reprogramming.^179^, ^180^

Functionally, TAMs mediate both immunostimulatory and immunosuppressive sequelae of RT. On the stimulatory side, low‐to‐moderate dosing can induce VCAM‐1 and TH1‐type chemokines in tumor vasculature, normalize vessels, and recruit CTLs, augmenting antitumor immunity.^181^ Conversely, hypoxic post‐RT TMEs favor immunosuppression via HIF‐1α‐dependent chemokine release, enhanced PD‐L1 expression on TAMs and tumor cells, and CD8^+^ T‐cell inhibition.^182^, ^183^ TAMs also influence RT outcomes through modulation of angiogenesis and matrix remodeling, and via paracrine induction of epithelial–mesenchymal transition in tumor cells through ICAM‐1/LFA‐1/Wnt3a/β‐catenin signaling.^184^

Given these divergent roles, therapeutic strategies seek to skew TAM populations toward an M1 phenotype or limit M2 recruitment. From a practical standpoint, RT is clinically accessible and readily tunable in dose and fractionation to engage immune‐active programs and combine with immunotherapy; however, its effects on TAMs are strongly regimen‐dependent, and hypoxia‐driven M2 recruitment together with normal‐tissue tolerance can limit durability and constrain parameter choices. Promising approaches include blockade of MIF–CD74, CD47–SIRPα, and CD24–Siglec‐10 axes in combination with RT, or addition of PD‐1/PD‐L1 inhibitors to overcome RT‐induced immune checkpoints. Targeting metabolic and signaling nodes such as CD73 or TGF‐β can synergize with RT to amplify cGAS‐STING–IFN signaling and curtail immunosuppressive TAM infiltration.^185^ Ultra‐high dose rate FLASH‐RT has shown distinct potential to overcome radio resistance, elevating M1/M2 ratios, reversing immune suppression, and enhancing CD8^+^ T‐cell–M1 TAM synergy in preclinical models.^186^ Despite these advances, key translational parameters—RT dose, fractionation, and combination scheduling with checkpoint blockade or myeloid‐targeting agents—remain incompletely defined, underscoring the need for systematic in vivo mapping of TAM polarization dynamics under varied RT regimens.

## 5 CONCLUSION AND FUTURE PERSPECTIVE

TAMs are pivotal regulators of the TIME, exerting either potent anti‐tumor effects or supporting tumor progression depending on their polarization states. Recent advances suggest that NIPS strategies—including ultrasound, optical‐based modalities, electromagnetic and electrical stimulation, and IR—provide precise and repeatable approaches to modulate TAM phenotypes and reshape the tumor immune landscape. By engaging distinct biophysical mechanisms, such as mechanical stress, ROS generation, hyperthermia, and ICD, NIPS interventions can promote reprogramming toward pro‐inflammatory, M1‐like macrophage programs and amplify antitumor immunity, potentially reducing reliance on sustained systemic pharmacological exposure.

However, despite encouraging preclinical evidence, clinical translation of NIPS for TAM modulation remains at an early stage. A key practical consideration is that the clinical impact of NIPS is ultimately governed by where and how reproducibly energy can be delivered, and whether immune‐active regimens can be implemented within a definable normal‐tissue tolerance window. Accordingly, the “best‐fit” NIPS modality is often determined less by a single mechanistic pathway than by lesion accessibility (superficial/endoluminal vs. deep‐seated), tissue attenuation and focusing, baseline hypoxia/perfusion and stromal density, proximity to critical structures, and compatibility with standard‐of‐care workflows. In this context, optical modalities are naturally positioned for superficial or endoluminal settings with high local controllability; accordingly, clinical salvage PDT using talaporfin sodium has been evaluated for locally failed esophageal cancer after chemoradiotherapy/RT, supporting the feasibility of endoluminal light‐based intervention in anatomically accessible lesions.^187^ Ultrasound‐based approaches are attractive for deep‐seated tumors due to penetration and image‐guided focusing; notably, focal HIFU has progressed into prospective multicenter studies for localized prostate cancer, illustrating how acoustic focusing can be operationalized in clinically relevant deep‐tissue targets.^188^ Electrical‐field‐based strategies offer programmable bioelectrical perturbation in anatomically feasible settings; in practice, TTFields has been clinically deployed in glioblastoma with pivotal randomized evidence, providing a clear precedent for noninvasive, field‐based therapy in solid tumors.^189^ By comparison, magnetic platforms offer remote controllability and deep penetration but typically require reliable intratumoral material deposition to achieve consistent biological output, whereas RT remains a clinically scalable backbone with standardized planning, even though its immunological outputs are strongly regimen‐dependent.^190^, ^191^, ^192^

Equally important, advancing NIPS‐based TAM targeting requires rigorous biosafety evaluation. Although NIPS avoids systemic drug distribution, it can still induce modality‐specific normal‐tissue effects, and combination regimens may introduce additional risk through amplified local or systemic inflammation.^193^ Therefore, defining immune‐effective yet tolerable parameter windows—supported by standardized dosimetry, appropriate monitoring, and clinically meaningful assessment of both acute and late tissue responses—will be essential for reproducible and safe translation.^193^, ^194^

Future development of this field will require integrated multidisciplinary efforts to clarify the molecular underpinnings of NIPS‐induced immune modulation, reduce heterogeneity in stimulation parameters, and address variability across tumor types and patient populations. Priority directions include establishing harmonized reporting standards, incorporating imaging‐enabled guidance and monitoring where applicable, identifying clinically actionable immune endpoints linked to TAM dynamics, and designing rational combinatorial regimens with established immunotherapies to achieve synergistic and durable therapeutic outcomes. With continued mechanistic exploration, technological refinement, and clinical validation, NIPS‐based TAM reprogramming may become a transformative component of next‐generation cancer immunotherapy and expand the scope of precision and personalized oncology.

## AUTHOR CONTRIBUTIONS

**Tingyu Zhang and Jie Lan:** Literature search and data analysis; writing – original draft preparation; writing – review and editing. **Wangrui Peng:** Literature search and data analysis. **Huai Yang and Yiyang Huang:** Literature search and data analysis. **Meng Du and Linyuan Jin:** Conceptualization; writing – review and editing. **Zhiyi Chen:** Funding acquisition; supervision. All authors contributed and approved the submitted version.

## FUNDING INFORMATION

This work was supported by National Natural Science Foundation of China (82572257, 82272028), Health Research Project of Hunan Provincial Health Commission (W20241010), Hunan Provincial Health High‐Level Talent Scientific Research Project (R2023010), and Hunan Provincial Innovation Foundation for Postgraduate (CX20251476).

## CONFLICT OF INTEREST STATEMENT

The authors declare no conflict of interest.

## CONSENT FOR PUBLICATION

All authors agreed with the content of this manuscript and approved the submitted version.

## Data Availability

Data sharing not applicable to this article as no datasets were generated or analyzed during the current study.
